# A Critical Advantage of Hypnobirthing to Ameliorate Antenatal Depression: A Systematic Review and Meta-Analysis

**DOI:** 10.3390/healthcare13070705

**Published:** 2025-03-23

**Authors:** Feni Betriana, Sunarno Sunarno, Wiwit Nurwidyaningtyas, Amelia Ganefianty

**Affiliations:** 1 Center for Biomedical Research, National Research and Innovation Agency (BRIN), Cibinong 16911, Indonesia; suna029@brin.go.id (S.S.); wiwi026@brin.go.id (W.N.); 2 Department of Nursing, Hasan Sadikin General Hospital, Bandung 40161, Indonesia; ganefianty@gmail.com

**Keywords:** depression, hypnobirthing, hypnotherapy, hypnosis for birth, systematic review, pregnant women

## Abstract

Background: Pregnant women are likely to experience depression due to various factors. Hypnobirthing is a non-invasive therapy that can be used to treat depression during pregnancy. This study aims to systematically review the effect of hypnobirthing on antenatal depression. Methods: This study employs a systematic review following PRISMA guidelines. Articles were retrieved from PubMed, ScienceDirect, the Cochrane Library, Google Scholar, and hand searches. Articles were included for review if they met the following inclusion criteria: (1) published in the last ten years (2014–2024); (2) the articles published in English; (3) article types are original research articles and reviews; (4) the full text can be retrieved; and (5) the findings of the selected articles should discuss the effect of hypnobirthing or hypnosis for birth. Articles were critically appraised using Joanna Briggs Institute tools. Results: The initial search yielded 7603 records; nine studies met the inclusion criteria for systematic review, and four studies for meta-analysis. The articles were analyzed, and findings were presented in narrative ways and categorized into three themes: the effect of therapy, the administration of the therapy, and the mechanism of the therapy. The therapy was performed by different methods, involving hypnosis, daily relaxation, progressive muscle relaxation, breathing exercises, the J-breathing technique, meditation, visualization, and ego strengthening. Additionally, participants were encouraged to do self-practice at home and were followed up by personal visits, phone calls, or text messages. Conclusions: The majority of the studies found that hypnobirthing ameliorated antenatal depression, despite the variation in initial administration and the duration of therapy. Further research is needed to standardize therapy protocols and explore long-term outcomes.

## 1. Introduction

Women are likely to experience various mental health issues during pregnancy [1]. One of the most recognized mental health issues among pregnant women is depression [2]. Depression is a mood condition characterized by chronic sorrow and loss of interest [3]. According to a systematic review and meta-regression, the prevalence of perinatal depression is projected to reach 11.9% worldwide [4]. These cases can be even higher in various contexts and situations, such as the regions or the existence of a pandemic situation. The World Health Organization (WHO) reported that the cases of depression among pregnant women in developing countries reach 15.6% during pregnancy and 19.8% after childbirth [5]. Meanwhile, during the COVID-19 pandemic, depression during pregnancy increased in several countries, such as increasing to 25% in Belgium, to 35.4% in Turkey, to 19.5% in Sri Lanka, and to 11–29% in China [6]. Meanwhile, in Canada, 37% of 1987 pregnant women reported clinically relevant symptoms of depression during the COVID-19 pandemic [7].

Depression during pregnancy is caused by multiple factors, including biological, psychological, and social factors. From social factors, lack of social support during pregnancy is related to the feelings of despair, anxiety, and self-harm [2]. Having a history of mental health problems, an insecure financial condition of the family, lower academic attainment, being the victim of domestic violence, perinatal smoking or drinking, and multiparity are also reported to relate to perinatal depression [8]. From the biological aspect, one of the factors is linked to the deviation of the hypothalamic–pituitary–adrenal (HPA) axis molecular markers. Chronic stress can cause increased HPA axis activity, which is crucial in the pathophysiology of depression [9]. Exposure to prenatal stress in rodent research demonstrated a consistent dysfunction in the HPA axis, which may enhance the susceptibility to mental disorders [10].

Depression during pregnancy needs to be treated as early as possible. Untreated maternal depression has a positive correlation with stillbirth, preterm birth, low pregnancy age, poor birth weight, and maternal morbidity, involving perinatal problems, higher surgical delivery, and postpartum depression [11]. In severe cases, this mental burden might lead mothers to commit suicide [5].

To treat depression and other psychological problems among pregnant women, several pharmacological and non-pharmacological approaches have been employed. One of the non-pharmacological interventions that is gaining popularity is hypnobirthing. Hypnobirthing integrated self-hypnosis with visualization and breathing techniques, aiming for a comfortable childbirth [12]. The term hypnobirthing was coined by Mongan, who first applied this technique during the birth of her granddaughter in 1990 [13]. Hypnobirthing was then implemented widely to help women during birth.

Several studies have investigated the efficacy of hypnobirthing or hypnosis for birth for various outcomes for mothers and children, with many of them focused on alleviating fear of childbirth and anxiety [13,14,15]. Meanwhile, studies exploring its effectiveness on depression are scarce, with the variations in the therapy administration in terms of methods, number, and length of sessions. These variations might result in the lack of consensus regarding hypnobirthing protocol. Within this scarce literature, a systematic review [16] was found to explore the psychological impact of hypnosis for pregnancy and childbirth. However, based on its objective, that systematic review focused not only on depression but also on other psychological impacts, including anxiety and fear of birth. Another systematic review [17] was also identified to explore the impact of hypnotherapy but focusing on fear, pain, and the birth experience. Meanwhile, studies that systematically review the effect of hypnobirthing on depression among women during pregnancy have not been found to date. Clarifying the effectiveness of hypnobirthing on depression, the therapy administration, and the mechanism of the therapy through a systematic review is fundamental as a source of information for guiding the management of antenatal depression through hypnobirthing. Therefore, this study aims to systematically review the effect of hypnobirthing to decrease depression among pregnant women.

## 2. Materials and Methods

### 2.1. Research Question

The research question in this systematic review is as follows: How is the effect of hypnobirthing on antenatal depression?

This research question is derived from the following PICO:
PPopulationPregnant womenIInterventionHypnobirthing or hypnosis for birthCComparisonUsual careOOutcomeDepression


In that PICO, usual care refers to routine antenatal care provided by the healthcare facilities, such as routine pregnancy checks and baby development, receiving advice on a healthy diet, counseling by an obstetrician regarding perceived complaints, or learning back massage to make pregnant women relax.

### 2.2. Study Design

The design of this study is a systematic review that evaluates studies describing the effect of hypnobirthing on depression among pregnant women. The protocol used in this systematic review follows the Preferred Reporting Items for Systematic Review and Meta-analysis (PRISMA) protocol [18].

### 2.3. Protocol Registration

The protocol of this systematic review was registered in the PROSPERO International prospective register of systematic reviews with registration number (CRD42024598822). 

### 2.4. Sources and Search Strategy

Articles were searched from reputable electronic databases, including PubMed, ScienceDirect, and Cochrane Library, along with relevant articles identified from Google Scholar and hand searches. Hand searches were performed by checking the references from the relevant articles.

The initial search used the following keywords: “hypnobirthing”, “hypnotherapy”, “hypnosis”, “depression”, “pregnant women”, and “pregnancy”, using the Boolean operators AND and OR.

### 2.5. Study Selection

Articles that fulfilled the inclusion criteria were included for this review. The inclusion criteria are as follows: (1) published in the last ten years (2014–2024); (2) the articles published in English; (3) article types are original research articles and reviews; (4) the full text can be retrieved; and (5) the findings of the selected articles should discuss the effect of hypnobirthing or hypnosis for birth on depression. Especially for studies with quantitative design, to be eligible for included studies, depression or depressive symptoms should be evaluated using validated tools, such as the Depression Anxiety Stress Scale-21 (DASS-21), the Edinburgh Postnatal Depression Scale (EPDS), etc. In addition, for original research articles with the intervention, the intervention given in the reviewed studies focused on providing hypnobirthing or hypnosis for birthing to help pregnant women feel confident and calm. Those interventions refer to hypnosis-based therapies, including hypnobirthing, self-hypnosis, autohypnosis, hypnotherapy, deep relaxation, breathing techniques, visualization, etc. In order to allow the researchers to fully comprehend the included studies that lead to satisfied analysis, only studies published in English were included in this systematic review. Meanwhile, the exclusion criteria include the following: (1) published before 2014; (2) non-English publication; (3) other than original articles and reviews; (4) duplicated articles; (5) full text cannot be retrieved; and (6) findings of the selected articles do not discuss the effect of hypnobirthing among pregnant women.

The initial screening for title and abstract was performed by the first author to decide if the study met the inclusion criteria. Further screening was performed in collaboration with another author in the research team. Study selection was conducted in November 2024 after the PROSPERO registration number was obtained.

### 2.6. Procedures for Data Extraction, Appraisal, Analysis, and Synthesis

The full text of the included articles was extracted, appraised, analyzed, and synthesized. Quality appraisal was critically performed using the Joanna Briggs Institute (JBI) appraisal tools. Two reviewers of the research team independently reviewed and appraised the articles. The potential score ranged from 0 to 13, based on the design of the study. Each item was assessed as “Yes”, “No”, “Unclear”, and “Not applicable”. The response with “Yes” was scored as 1. The quality of the articles was categorized based on the total scores. The quality of studies with scores less than 50% is considered weak; studies with scores of 50–70% are considered moderate, and studies with scores above 70% are deemed high quality. Any discrepancies between the two reviewers were resolved through discussion with all research team. The final selected articles were extracted using standardized data extraction of a matrix table that contains authors, year, design of the studies, participants, countries, the study’s main outcomes, and JBI scores. The outcomes of this systematic review were organized into three categories: (1) the effect of hypnobirthing on depression among pregnant women, (2) administration of the therapy, and (3) the mechanism of the therapy on depression. Despite the differences in study types, all results were merged in order to obtain an overall picture of how hypnobirthing was implemented and its effects on depression. These different study types provide different ways of presenting the results of hypnobirthing. For quantitative studies, the therapy was provided as an intervention between pre- and post-measurements of depression or depressive symptoms using validated tools. The effects of hypnobirthing were evaluated from the significant differences between the scores before and after intervention (please refer to Table 1). Meanwhile, the qualitative studies provide the descriptions of women’s experiences receiving hypnobirthing during pregnancy, including the feelings they had after participating in the therapy. By merging all results despite various study types, it is expected to provide insights for those who will implement hypnobirthing in the future. 

Moreover, we used a fixed-effect model for meta-analyses of outcomes reported by multiple studies that were sufficiently alike to justify combining results [19]. This meta-analysis assessed the impact of hypnobirthing on antenatal depression using data from two randomized controlled trials (RCTs) and two quasi-experimental studies. The analysis was conducted using RevMan V 5.4.1 software, employing the inverse variance method, a fixed-effect model, and continuous data synthesis. To ensure statistical consistency, heterogeneity was assessed using I^2^ < 50% and Chi^2^ < 0.10, with a 95% confidence interval [20].

## 3. Results

**Figure 1:**
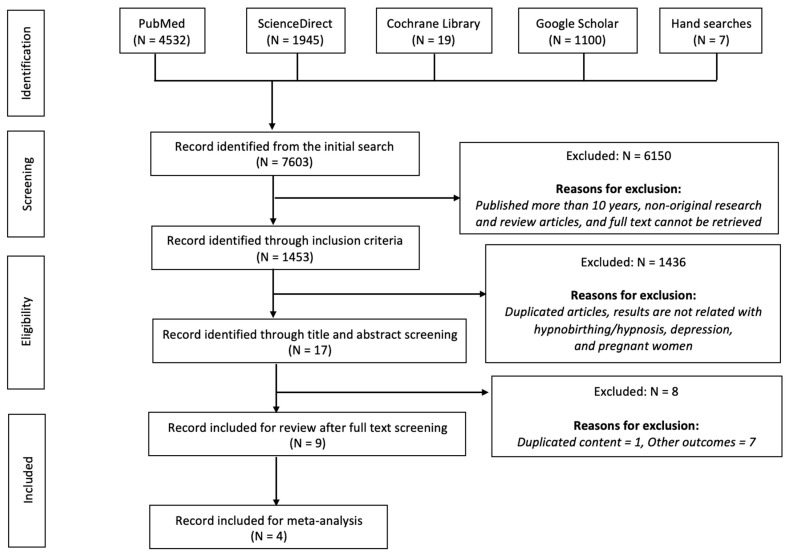
PRISMA flowchart.

An initial search using keywords from four databases along with hand searches yielded 7603 records. After screening for inclusion criteria, 6150 records were removed, and 1453 records were retained. Afterwards, 1453 were screened for titles and abstracts, which then excluded 1436 records due to duplicated articles, and the studies’ results were not in line with the outcomes of this systematic review. Next, title and abstract screening retained 17 articles that were further assessed for full text. Full text assessment removed 8 articles due to other outcomes (7 articles) and duplicate contents (1 article), and maintained 9 articles for systematic review. Four of them were then included for meta-analysis. The search flow is presented in Figure 1.

### 3.1. Characteristics of the Included Studies

The final full-text assessment included nine articles for review, with the total number of participants, including both experimental and control groups, being 377 participants. Those articles consist of two quasi-experimental studies [21,22], one systematic review [16], two randomized controlled trials [15,23], and four qualitative studies [24,25,26,27]. For original research articles, two studies were conducted in Malaysia [21,22], one study was conducted in Indonesia [23], one study was conducted in Norway [26], one study was conducted in Pakistan [15], one study was conducted in England [24], and one study was conducted in Scotland [25]. Among nine studies, eight studies are considered to have high-quality appraisal, and one study is deemed to have moderate-quality appraisal. The characteristics of each study are presented in Table 1.

**Table 1:** The characteristics of the included studies.

No.	Authors, Year	Study Design	Participants	Country	Main Study Description	JBI Scores
1.	Beevi et al., 2019 [22]	Quasi-experimental design	Pregnant women N = 28 (Experimental group) N = 28 (Control group)	Malaysia	▪ Results: The experimental group had lower postpartum depressive symptoms (M = 1.25, *p* < 0.002) and postpartum depression (M = 5.64, *p* < 0.001) compared to the control group (M = 6.32 for depressive symptoms, M = 10.64 for postpartum depression). ▪ Measurement tool: The Depression Anxiety Stress Scale-21 (DASS-21) was used to evaluate psychological symptoms, and the Edinburgh Postnatal Depression Scale (EPDS) was used to measure postpartum depression. ▪ Applied method: - Hypnosis was administered for four meetings, at weeks 16, 20, 28, and 36 of pregnancy. - Pregnant women were required to put their thumb and index finger at the same time and progressive muscle relaxation according to Hartland’s hypnotherapy training. They were suggested that the symptoms travel down to the thumbs and index finger before disappearing from the body. - Pregnant women received ego strengthening and were encouraged to practice self-hypnosis at home.	9/9
2.	Beevi et al., 2016 [21]	Quasi-experimental design	Pregnant women in their second trimester N = 28 (Experimental group) N = 28 (Control group)	Malaysia	▪ Results: The experimental group had lower depressive symptoms at 36 weeks of their pregnancy (M = 3.29, *p* = 0.012). ▪ Measurement tool: The depressive symptoms were measured using the Depression Anxiety Stress Scale-21 (DASS-21). ▪ Applied method: - The therapy comprised 4 sessions, one session at the baseline (week 16), followed by the next 3 points (weeks 20, 28, and 36). - The Hartland’s hypnotherapy training was adjusted for the ego-strengthening script. - Self-hypnosis was taught at session 1, removal of physical and psychological symptoms was provided in every session, and advice for a favorable experience of labor and postpartum was delivered at sessions 3 and 4. - Follow-ups for self-hypnosis practice at home were made during hypnosis sessions at every time point and via phone calls once a week until delivery.	9/9
3.	Catsaros and Wendland, 2023 [16]	Systematic review	N = 7 studies	Not specified	▪ Results: - Seven studies were included, with the total number of participants in the experimental groups being 1332 women. - The majority of included studies revealed that hypnosis had a beneficial impact on anxiety, despair, and dread of childbirth, motivating women with a greater feeling of comfort and strengthening the whole emotional experience. ▪ Inclusion criteria for the included studies: - Studies published in English from January 2000 to December 2021. - The type of studies is experimental studies, employing hypnosis for pregnant or postpartum women. - Studies examined depression symptoms or other psychological issues.	10/11
4.	Himawati et al., 2024 [23]	Randomized control trial	Pregnant women (N = 154)	Indonesia	▪ Results: Childbirth education accompanied by assistance with hypnobirthing reduced depression (effect size = 1.19, *p* < 0.001). ▪ Measurement tool: The Edinburgh Postnatal Depression Scale (EPDS). ▪ Applied method: - Hypnobirthing was provided together with childbirth education regarding pregnancy, labor, and physical and spiritual assistance for pregnant women. - Hypnobirthing was provided through a relaxation technique. - Therapy was given 2 times a week with a duration of 3 h.	7/13
5.	Uldal et al., 2023 [26]	Descriptive phenomenological study	Pregnant women (N = 9)	Norway	▪ Results: The therapy increased the confidence to give birth, developed the childbirth into a meaningful experience, and decreased the fear of birth and the pain of birth. ▪ Applied method: The therapy was provided through daily relaxation, writing notes with the positive affirmation, and practicing breathing techniques.	10/10
6.	Uludag et al., 2023 [27]	Phenomenological design	Hypnobirthing stories obtained from four blogs N = 13	Not specified	▪ Results: Hypnobirthing eliminated participants’ fears and made them ready for childbirth, relaxed them during the birth process, made them think positively about birthing, and made them feel calm and comfortable during childbirth. ▪ Applied method: - J-Breathing in hypnobirthing: inhale through the nose, send the breath to the womb, and slowly exhale through the mouth. - Turning down the light, listening to music, and performing relaxation.	8/10
7.	Yaqoob et al., 2024 [15]	Randomized control trial	Pregnant women N = 25 (Experimental group N = 25 (Control group)	Pakistan	▪ Results: Participants in the experimental group showed a lower risk of postpartum depression (M = 11.8, *p* < 0.001) than those in the control group (M = 14.5) with the first vaginal delivery one week after giving birth. ▪ Measurement tool: The Edinburgh Postnatal Depression Scale (EPDS). Inclusion criteria of participants: women who obtained a score of 13 or greater on the EPDS and a score between 7 and 9 on Templer’s Death Anxiety Scale. ▪ Applied method: - Hypnobirthing employs self-hypnosis, breathing practices, visualization, deep relaxation techniques, labor meditation techniques, and strategies to alleviate anxiety about death, painful labor, and postpartum depression. - The experimenter made home visits and phone calls for 4 weeks to observe the whole experiment.	11/13
8.	Finlayson et al., 2015 [24]	Qualitative interviews	N = 19 16 first-time mothers and 3 birth companions	England	▪ Results: The majority of participants expressed beneficial experiences of self-hypnosis, emphasizing feelings of relaxation, confidence, and empowerment about the upcoming birth; reduced feelings of worry; and reduced feelings of anxiety. ▪ Applied method: - The therapy included two antenatal self-hypnosis training sessions and a CD for the participants to listen to every day from 32 weeks till birth. - Participants were encouraged to listen to the CD for 25 min each day for 7–10 weeks. - The training began with an explanation of the physiology of labor and birth, with the aim of providing a foundation before moving on to self-hypnosis training.	9/10
9.	Tabib et al., 2024 [25]	Descriptive qualitative study	N = 26 17 women and 9 partners	Scotland	▪ Results: Participants reported feeling confident, prepared, and excited about the approaching birth. Increased confidence was found to reduce negative feelings such as dread and worry, as well as increase favorable views about birthing. ▪ Applied method: - Hypnosis was provided in a single antenatal relaxation class (ARC) along with breathing, visualization, and relaxation. - Relaxation caused a state of awareness that differed from what one encounters in everyday life. Participants in this state reported feeling focused, relaxed, mindful, and attentive.	8/10

### 3.2. Outcome Categories of the Included Studies

The outcomes of this systematic review were organized into three categories, which are (1) the effect of the therapy, (2) administration of the therapy, and (3) the mechanism of the therapy.

#### 3.2.1. The Effects of the Therapy

The effects of the therapy revealed from the reviewed studies include lowering the risk of postpartum depression [15], lowering postpartum depressive symptoms and postpartum depression [21,22], and reducing depression [23]. The changes in depression and depressive symptoms were evaluated before and after the intervention using validated tools such as the Depression Anxiety Stress Scale-21 (DASS-21) and the Edinburgh Postnatal Depression Scale (EPDS).

In Beevi et al.’s [22] study, the hypnosis interventions were provided to pregnant women for four sessions. Before and after the intervention, depression was measured using the Depression Anxiety Stress Scale-21 (DASS-21). After four sessions of the therapy, the mean scores of depressive symptoms reduced [22]. Similar to that study, another study [21] provided hypnosis therapy for pregnant women for four sessions and compared them with another group without the therapy. Before and after the intervention, psychological symptoms and postpartum depression were assessed using DASS-21 and EPDS, respectively. Findings after the therapy showed that pregnant women receiving the therapy showed lower depressive symptoms (M = 1.25) compared to those who did not receive hypnosis (M = 6.73) [21].

Hypnobirthing, given along with childbirth education, also proves effective in reducing depression among pregnant women, indicated by the effect size = 1.19 and *p* < 0.001 [23]. In a randomized controlled trial [15], participants in the control group had a greater chance for postpartum depression than those in the experimental group who had their first vaginal birth a week after labor. Those assigned to the experimental group had a lower mean score of depression than those in the control group. Still, in that study, several techniques were employed by participants in the experimental group in order to relieve their symptoms more successfully, making hypnobirthing training a catalyst that significantly lowers postpartum depression, death anxiety, and labor pain [15]. Additionally, a systematic review [16] discovered that hypnosis had a favorable effect on improving the entire emotional experience, empowering women with a greater sense of confidence, and reducing depression, anxiety, and birth-related fear.

Besides alleviating depression, hypnobirthing, or hypnosis for birth, also triggered positive feelings and emotions for pregnant women, including increasing the confidence to give birth and developing childbirth into a meaningful experience [26]. Hypnobirthing also helped mothers to feel ready for childbirth by creating positive thinking about birthing and relaxing themselves during the birth process [27].

Likewise, it is also reported that most women conveyed favorable experiences with self-hypnosis, emphasizing feelings of tranquility and empowerment, feeling more confident about the upcoming birth, reduced feelings of worry, and being able to control feelings of anxiety [24]. In line with those studies, another study [25] found that hypnosis given in the antenatal relaxation classes, along with breathing and relaxation, shifted negative emotions toward positive feelings of confidence, preparedness, empowerment, and excitement about the upcoming birth.

**Figure 2:**
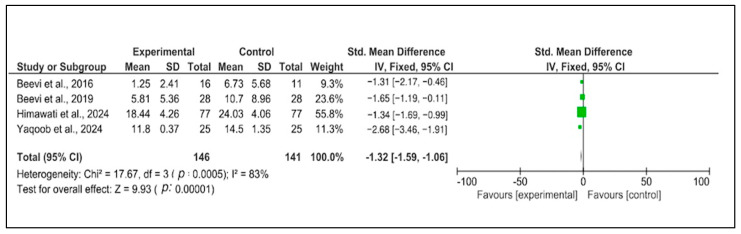
Forest plot. Evaluation of the ameliorate antenatal depression after hypnobirthing versus the control group used RevMan 5.4.1. [15,21,22,23].

Furthermore, significant heterogeneity was found between studies (*p* = 0.0005, I^2^ = 83%) for the analyzed outcome, so the fixed-effects model was used to summarize the mean effect size. The results indicated that the intervention led to a significant reduction in the measured outcome (SMD = −1.32, 95% CI = −1.59 to −1.06, *p* < 0.00001) (Figure 2). Due to high between-study heterogeneity, no subgroup analysis was performed. 

#### 3.2.2. Administration of the Therapy

Reviewed studies described various methods of therapy administration. These variations include the number of sessions, types of techniques, and follow-up manners. In Finlayson et al.’s study [24], the therapy was provided for two sessions from week 32 of gestation until the birth. The therapy was started by providing the description of the physiology of labor and birth to participants and was intended to provide basic instruction. Afterwards, the participants were requested to listen to the CD for 25 minutes each day for 7–10 weeks. The CD can be listened to at night before sleep or at any convenient time.

In another study [15], the hypnobirthing program was administered for three sessions from week 33 of gestation for the duration of four weeks. The therapy in that program comprised self-hypnosis, visualization, deep relaxation techniques, labor meditation techniques, breathing exercises, and techniques for dealing with labor pain, death anxiety, and postpartum depression. The psychoeducation and management for pain, and death anxiety were offered at the first and second sessions. The third session discusses the labor meditation therapies used throughout the delivery process in the final week before childbirth. It focuses on shifting the sense of pain and replacing it with emotions of relaxation and control. To assist participants in following the therapy, a hypnobirthing training guide in the form of a booklet with four charts was provided for them. Home visits and phone calls were made by the experimenter to monitor the whole experiment [15].

Furthermore, the hypnosis therapy was provided in four sessions during pregnancy, starting from week 16 [21,22]. In that study [22], pregnant women were asked to put their index finger and thumb at the same time, and they were given progressive muscle relaxation based on Hartland’s hypnotherapy training. Afterwards, participants were given suggestions that symptoms travel down the arms to the thumbs and index fingers before leaving the body. They also received ego strengthening and were suggested to practice self-hypnosis at home in the evening before sleep. Furthermore, participants were followed up with via phone calls and text messages. At the follow-up sessions, they were reminded to practice self-hypnosis [22].

Similarly, in another study [21], the therapy was administered in four sessions. Training in self-hypnosis was given at the first session. Removal of physical and psychological symptoms was provided in all sessions. The third and fourth sessions contained suggestions for a pleasant labor and postpartum experience, including a close connection between mother and child and improved maternal psychological well-being. At the end of every session, participants were advised to practice self-hypnosis at home. Follow-ups were made through phone calls once a week until they were hospitalized for labor.

In Himawati et al.’s [23] study, hypnobirthing was provided along with childbirth education. The contents of childbirth education were related to education about pregnancy and childbirth, physical, psychological, and spiritual assistance. Hypnobirthing was provided by relaxation techniques two times a week with a duration of three hours. Meanwhile, in Uldal et al.’s study [26], the participants informed that they practiced hypnobirth through daily relaxation, writing notes with the positive affirmation, and practicing breathing techniques. In Uludag et al.’s study [27], hypnobirthing was practiced through J-Breathing by inhaling through the nose, sending the breath to the womb, and slowly exhaling through the mouth. In addition, hypnobirthing was also practiced by turning down the light, listening to music, and performing relaxation [27].

#### 3.2.3. The Mechanism of the Therapy

The majority of the studies do not discuss in detail how hypnobirthing decreased depression among pregnant women. Uludag et al. [27] highlighted that hypnobirthing prepared women for childbirth by making them feel calm and comfortable. In their study, hypnobirthing was provided by breathing exercises, J-Breathing, practicing positive thoughts about birthing, and childbirth education. It is believed that words and thought have power [28]. Positive thinking reduces stress hormones and results in less perceived stress [27].

Tabib et al. [25] explained that recognizing how maternal emotions affect delivery physiology was seen as one of the primary factors in the emotional transformation. This idea was regarded as a revelation, something that made sense and gave reason and meaning to the difficulties of labor. Meanwhile, Yaqoob et al. [15] explained that hypnobirthing uses deep relaxation techniques, breathing exercises, visualization, self-hypnosis, labor meditation, and techniques that target labor pain, postpartum depression, and death anxiety. Its main goal is to alter how pain is perceived and substitute it with sensations of control and relaxation. The method highlights how to use the body’s innate birthing instincts and the strength of the mind–body connection. Regular relaxation techniques and positive affirmations are used to practice and condition the mind. Women can achieve deep relaxation using hypnobirthing techniques, which can assist in lowering stress hormone release and increasing endorphin production—the body’s natural painkillers. It guarantees a more peaceful and natural delivery. Table 2 presents the summary of the therapy administration from the reviewed studies.

## 4. Discussion

Depression is generally caused by biological vulnerability in line with long-term stressful life events. The altered function of brain regions important to the stress response, including hypothalamic–pituitary–adrenal (HPA) axis hyperactivity, has been consistently found in individuals with depression [9]. Interestingly, both currently depressed and remitted patients exhibit higher stress levels and cortisol responses compared to healthy controls, indicating that HPA axis dysregulation may persist even after remission [29,30]. Various stress management techniques, including breathing exercises, meditation, guided imagery, and mindfulness, have been declared effective in treating stress-related disorders such as anxiety, depression, and post-traumatic stress disorder [31]. These findings highlight the importance of addressing both psychological and physiological aspects of the disorder.

This study aims to systematically review the effect of hypnobirthing on depression among pregnant women. In general, findings show that hypnobirthing, or hypnosis for birth, is effective in decreasing depression among pregnant women during pregnancy and after birth, as well as other psychological symptoms, such as anxiety, fear towards birth, and pain of birth. Besides improving negative emotions, hypnobirthing also triggered positive emotions, including feeling prepared and confident for birthing by developing a positive thought about birthing. Positive emotions play a crucial role in maternal-infant bonding and infant growth. Research indicates that mothers’ ability to recognize and respond to positive emotions in their infants is particularly important for developing sensitive parenting behaviors and fostering healthy child development. A study found that mothers’ ability to detect happiness specifically in their newborns’ facial expressions predicted greater observed maternal sensitivity four months later [32]. This suggests that recognizing positive emotions in infants may be uniquely important for promoting sensitive caregiving in low-stress parent-infant interactions. Additionally, fathers’ supportive responses to children’s positive emotions in childhood were indirectly associated with greater life satisfaction in young adulthood through increased positive emotional experiences [33].

The reviewed studies showed variations in the terminology used for the therapy given to the participants, which are hypnosis [16,21,22,24,25], hypnobirthing [15,23,27], and hypnobirth [26]. Even though those studies used different terms for their intervention, those interventions mainly refer to the same main therapy, which is hypnosis-based therapy for birth. According to the American Psychological Association [34], hypnosis is a process, or a condition stimulated by that process, where advice is employed to induce improvements in emotion, feelings, cognition, perception, or control over motor behavior. In terms of psychotherapeutic intervention, hypnosis is also referred to as hypnotherapy. Meanwhile, in the field of obstetrics, hypnosis used to prepare women for childbirth is known as hypnobirthing [17] or the Mongan method [28].

The included articles showed various ways of hypnobirthing administration. The frequency of official therapy with the trainers varied from two sessions [24] to three sessions [15] and four sessions [21,22]. The earliest therapy started from week 16 of pregnancy [21,22], then week 32 [24] and week 33 of pregnancy [15]. The therapy was provided for the duration of four weeks [15] to 7–10 weeks [24]. Apart from official therapy training with the trainers, participants were also encouraged to do self-practice at home, which was followed up by personal visits to participants’ houses, phone calls, or text messages [15,21,22].

In terms of the methods of administration, some studies [21,22] adopted Hartland’s hypnotherapy method, while another study mentioned J-breathing in hypnobirthing [27]. Other studies also combined the therapy along with other supporting interventions, such as writing notes with the positive affirmation [26], psychoeducation [15], and childbirth education [23]. Even though there are variations in the therapy administration, the main techniques refer to hypnosis-based techniques.

How hypnobirthing reduces depression can be explained by several possible mechanisms. From the mind–body approach, hypnobirthing provides women a comprehensive understanding of labor and how to ensure a positive birth experience, regardless of the outcome [26]. This made them feel relaxed and mentally prepared for birth, therefore anticipating the depression and other psychological conditions. Furthermore, it is argued that hypnobirthing techniques allow women to enter a profound state of relaxation, which can assist in minimizing the production of stress hormones and increasing the endorphin production, the body’s natural pain reliever [15].

Understanding the various risk factors, including childhood traumatic events, and utilizing appropriate assessment tools can lead to better outcomes for mothers, partners, and infants affected by antenatal or postnatal depression. Furthermore, assessing depression severity is crucial for appropriate treatment and support; in that manner, regular screening of both mothers and partners is recommended for a further holistic approach [35]. From an ethical standpoint, hypnobirthing aligns well with the principles of beneficence and patient autonomy. By providing women with tools to manage their stress and anxiety during childbirth, it empowers them to have more control over their birthing experience. However, it is crucial that healthcare providers obtain informed consent and respect women’s choices regarding depression management methods. Additionally, as with any medical intervention, practitioners must ensure that hypnobirthing techniques are applied safely and in conjunction with standard medical care to uphold the principle of non-maleficence [36].

Following the objective of this review, which is to review the effect of hypnobirthing on depression among pregnant women, studies included in this review involved women with depression. One of those studies [15] even included minors (under 18 years old) as the participants, which made them considered vulnerable groups. Therefore, ethical aspects are crucial considerations in implementing hypnobirthing for this group. In the included studies [15,23], participants were fully informed regarding the nature of the study, and study ethics were carefully handled throughout the research process. It was also reported that participants were allowed to participate in the therapy trial before the program began in order to make them feel secure with the therapy and that the therapy would not harm their pregnancy. The absence of hypnobirthing intervention provided for the control group, despite the existence of the depression or depressive symptoms, is another ethical concern. This has been discussed in the included studies. In some studies [21,22], hypnosis intervention was first offered to all participants, and those who declined to participate were assigned to the control group, while those who were willing to participate in the hypnosis intervention were assigned to the intervention group. Nonetheless, those in the control group still received usual routine antenatal care. Meanwhile, in another study [15], participants in the control group received placebo intervention in terms of reading a parenting book based on the prescribed regimen, similar to how the intervention group received hypnobirthing.

The outcome of this study implies that hypnobirthing in antenatal care might im-prove healthcare outcomes by reducing depression and other psychological symptoms among pregnant women, but a solid conclusion cannot be drawn. Moreover, since this review only included studies published in English, the transferability of these findings is limited, as they might not inform how hypnobirthing is practiced in other parts of the world. Some settings might have different cultural and socioeconomic situations as well as specific protocols and policies regarding antenatal care checkups. Therefore, the practice of hypnobirthing should be adjusted to the situation of each setting.

While hypnobirthing has been used to alleviate psychological symptoms during pregnancy and labor, such as depression, anxiety, fear of birth, etc., studies examining the effects of hypnobirthing on long-term outcomes, including maternal-infant bonding and child development, are scarce. Future research investigating the long-term outcomes of hypnobirthing is recommended.

### Strengths and Limitations of the Study

Since systematic literature regarding hypnobirthing for depression is scarce, this systematic review is expected to clarify the effect of hypnobirthing on maternal depression and provide insightful information for healthcare professionals regarding the implementation of hypnobirthing, which becomes the strength of this study. Moreover, this review includes studies from various countries and continents, implying the wider chances of this therapy being implemented generally to patients, populations, and communities with different backgrounds.

Nonetheless, some limitations were highlighted, including the limitation of the articles only published in the last ten years and only including articles published in English, which might limit the scope to access more articles. However, this duration was chosen as a way in order to obtain the recent articles; therefore, the state of the art of the therapy can be identified. Notably, the most appropriate time to begin hypnobirthing and the length of sessions required to control antenatal depression cannot be concluded from this review due to differences in study design and instruments for validating depression levels in the reviewed articles. Further research needs to apply depression scale measurements at several time points as a benchmark for determining the best methods for administering hypnotherapy for antenatal depression treatment. The consequences of hypnobirthing in the long run and its efficiency among other populations, for example, people with chronic or terminal illnesses, need to be further examined.

In addition, there are significant differences between the studies included in this review in terms of types of studies, participants, the applied method of hypnobirthing, duration of treatment, number of sessions administered, gestational age when the treatment started, follow-up methods, and evaluation of depression symptoms. There is a lack of use of validated tools to evaluate the changes in depression symptoms, making it difficult to draw a solid conclusion from this systematic review.

## 5. Conclusions

The findings of this study indicates that hypnobirthing in antenatal care might improve healthcare outcomes by reducing depression and other psychological symptoms among pregnant women. Findings show various methods for therapy administration, from two to four sessions during pregnancy, with the earliest starting from week 16 of pregnancy and continuing for around four to ten weeks until birth. Hypnobirthing can also be provided along with other supporting interventions, such as childbirth education and writing notes with self-positive affirmation. Nonetheless, the included studies in this review did not report in detail if and how hypnobirthing decreased depression among pregnant women. Future research exploring the path of how hypnobirthing decreases depression, especially from the pathophysiology point of view, is highly suggested.

## Figures and Tables

**Table 2:** Summary of the therapy administration from the reviewed studies.

No.	Summary
1.	The therapy was provided in two to four sessions during pregnancy.
2.	The earliest therapy was provided at week 16 and continued until birth.
3.	Along with the main hypnosis-based techniques, the therapy sessions also include relaxation, ego strengthening, breathing techniques, writing positive affirmations, and childbirth education.
4.	Apart from the official training session with the trainer, the participants were encouraged to do self-practice at home.
5.	Self-practice was performed every day for 25 min or two times a week for 3 h.
6.	To engage participants for self-practice, participants were followed up with a personal home visit, phone call, or text message.

## Data Availability

Data relevant to this study are included in the article.
